# Kono-S Anastomosis in Pediatric Crohn’s Disease: Experience at a Tertiary Referral Center

**DOI:** 10.3390/jcm14207403

**Published:** 2025-10-20

**Authors:** Valeria Dipasquale, Claudio Romano, Carmelo Romeo, Pietro Impellizzeri, Angela Simona Montalto, Giuseppe Navarra

**Affiliations:** 1 Pediatric Gastroenterology and Cystic Fibrosis Unit, Department of Human Pathology in Adulthood and Childhood “G. Barresi”, University Hospital “G. Martino”, 98124 Messina, Italy; dipasquale.valeria@libero.it; 2 Pediatric Surgery Unit, Department of Human Pathology in Adulthood and Childhood “G. Barresi”, University Hospital “G. Martino”, 98124 Messina, Italy; romeoc@unime.it (C.R.); pietro.impellizzeri@unime.it (P.I.); asmontalto@unime.it (A.S.M.); 3 Surgical Oncology Division, Department of Human Pathology in Adulthood and Childhood “G. Barresi”, University Hospital “G. Martino”, 98124 Messina, Italy; gnavarra@unime.it

**Keywords:** Kono-S anastomosis, Crohn’s disease, children

## Abstract

**Objectives:** The Kono-S anastomosis is a bowel-sparing surgical technique developed to reduce postoperative recurrence in Crohn’s disease (CD). While its efficacy has been established in adults, data in pediatric populations remain scarce. This study aims to evaluate the safety, feasibility, and early outcomes of the Kono-S technique in children and adolescents with CD at a single tertiary referral center. **Methods:** A retrospective review was conducted of pediatric CD patients who underwent bowel resection with Kono-S anastomosis between January 2022 and March 2025. Data collected included patient demographics, surgical indications, intraoperative findings, postoperative complications, and follow-up, including endoscopic surveillance. **Results:** Eleven patients (median age 14.7 years; 63.6% female) underwent laparoscopic Kono-S anastomosis, primarily ileocolic. Indications included stricturing disease (n = 6), intra-abdominal abscesses (n = 3), or both (n = 2). No postoperative complications occurred. Median follow-up was 21 months. Follow-up endoscopy was performed in nine patients: eight had a Rutgeerts score of 0, and one had a score of 1. All patients began anti-TNF-alpha therapy within a median of 10.4 weeks post-surgery. **Conclusions:** This first Italian pediatric case series suggests that Kono-S anastomosis may be safe and feasible in CD, with low early endoscopic recurrence. Larger, multicenter studies with long-term follow-up are needed to validate these findings.

## 1. Introduction

Kono-S anastomosis, a handsewn, antimesenteric, functional end-to-end technique, was developed to reduce the risk of anastomotic recurrence following intestinal resection for Crohn’s disease (CD). First introduced in adult patients by Kono et al. in 2011, the technique aims to address the limitations of conventional anastomotic approaches by strengthening the anastomotic line with a supporting column, which preserves lumen thickness and minimizes mesenteric inflammation [1].

In adult populations, accumulating evidence has supported the safety and efficacy of the Kono-S technique. Several studies, including prospective trials and meta-analyses, have demonstrated lower rates of surgical and endoscopic recurrence when compared to standard stapled side-to-side anastomoses [2,3,4]. The procedure consists of (a) performing an antimesenteric functional endoscopic anastomosis by hand sewing of the mesenteric edges to maintain the shape of the lumina and to prevent narrowing, and (b) using longitudinal anastomosis to facilitate the anastomosis from side to side [5].

Despite these promising results, pediatric applications remain limited. To date, only two retrospective pediatric studies have evaluated Kono-S in this context [6,7]. A recent report found that patients who received Kono-S anastomosis had shorter hospital stays and fewer postoperative symptoms compared to those with conventional anastomoses [6]. Another study showed that 60% of pediatric patients achieved endoscopic remission at six months postoperatively [7]. In pediatric patients, it offers a promising approach because children often face a lifelong disease course, so reducing recurrence risk early on is crucial. The present study reports our institution’s experience with the Kono-S anastomosis in a pediatric cohort, contributing the longest median follow-up with endoscopic assessment at 12 months, which was missing in the prior pediatric reports.

Pediatric Crohn’s disease presents unique challenges compared with adult disease, including an earlier age of onset, a more aggressive phenotype, and the risk of impaired growth and delayed puberty due to chronic inflammation and repeated surgical interventions. Approximately 20–30% of children with CD require intestinal resection within 10 years of diagnosis, and up to half of these may require further operations during adolescence or early adulthood [8,9]. Anastomotic recurrence is particularly problematic in pediatrics because it compounds the risk of multiple resections, bowel shortening, nutritional compromise, and long-term morbidity. Consequently, strategies to minimize recurrence are especially valuable in this population.

Surgical recurrence in CD is thought to be driven by residual mesenteric inflammation, ischemic injury at the suture line, and altered luminal flow dynamics. The Kono-S technique is designed to mitigate these factors by creating a broad, antimesenteric anastomotic column that resists narrowing and minimizes mesenteric traction [1]. This design may be particularly advantageous in children, where bowel diameter is smaller and risk of luminal compromise is proportionally greater. The preservation of luminal caliber and prevention of kinking are critical in ensuring adequate bowel function throughout childhood and adolescence.

Several large adult series and systematic reviews have consolidated the benefits of the Kono-S technique. In a 2022 meta-analysis of more than 800 patients it was found that the Kono-S approach was associated with significantly lower rates of endoscopic recurrence and reoperation compared with stapled anastomoses [2]. Another research paper similarly demonstrated reductions in anastomotic recurrence, particularly at the 24-month timepoint [4]. These findings have stimulated interest in translating the procedure to pediatrics, where the relative gains may be even greater due to the longer life expectancy and cumulative surgical risk.

Beyond recurrence, pediatric-specific considerations include postoperative quality of life, psychosocial impact, and hospital stay. Minimizing complications and ensuring rapid recovery are paramount, as these factors affect schooling, growth, and social development. A technique that reliably reduces recurrence and avoids complications could alter the natural course of CD in children by delaying or preventing multiple resections. Nevertheless, published pediatric experience with Kono-S remains sparse, consisting of only two small retrospective studies [6,7]. These gaps highlight the need for further investigation in larger, well-characterized cohorts.

This study, therefore, aims to contribute additional real-world data on Kono-S anastomosis in children with CD. Specifically, we report a pediatric series with a median follow-up exceeding 20 months and a prospective endoscopic assessment at 12 months.

## 2. Materials and Methods

### 2.1. The Study Design and Setting

We conducted a retrospective chart review of all pediatric patients diagnosed with Crohn’s disease (CD) who underwent bowel resection with a Kono-S anastomosis between January 2022 and March 2025 at a tertiary referral centre specializing in pediatric inflammatory bowel disease. This center functions as a regional hub for complex pediatric IBD cases, allowing the consolidation of experience with advanced surgical techniques and providing comprehensive multidisciplinary care. The Kono-S was adopted as part of evolving routine clinical practice for pediatric Crohn’s resections from 2022 onwards. The study protocol was approved by the institutional ethics board, ensuring that all research activities conformed to ethical standards for studies involving children, including protection of patient privacy and confidentiality. Written informed consent was obtained from parents or legal guardians in all cases, and assent was sought from patients where appropriate, in accordance with national regulations. Patient confidentiality was strictly maintained; all data were anonymized before analysis.

### 2.2. Patient Selection

Inclusion criteria were as follows: (i) confirmed diagnosis of Crohn’s disease based on the revised Porto criteria [10], (ii) age less than 18 years at the time of surgery, and (iii) a minimum postoperative follow-up of three months to allow for adequate early outcome assessment. Patients with prior ileocecal resections for CD or who required reoperation during follow-up were included to reflect the typical pediatric CD population, which often experiences multiple surgical interventions over time. Exclusion criteria were (i) patients with indeterminate colitis, (ii) patients undergoing bowel resection for indications other than Crohn’s disease, and (iii) patients with follow-up shorter than three months. By applying these criteria, we ensured that our analysis focused specifically on pediatric Crohn’s disease and minimized potential confounding from other intestinal disorders. All eligible patients during the study period were included consecutively. No cases were excluded beyond the predefined criteria.

Demographic, clinical, and surgical variables were systematically retrieved from electronic medical records by two independent reviewers. This dual-review process was implemented to enhance data accuracy and reduce errors commonly associated with retrospective studies. Extracted variables included age, sex, disease phenotype, prior surgical history, medication use, indication for surgery, and anatomical site of resection. Collecting this comprehensive dataset allowed for detailed characterization of the patient cohort and provided context for interpreting surgical and postoperative outcomes.

### 2.3. Surgical Technique and Postoperative Management

**Figure 1:**
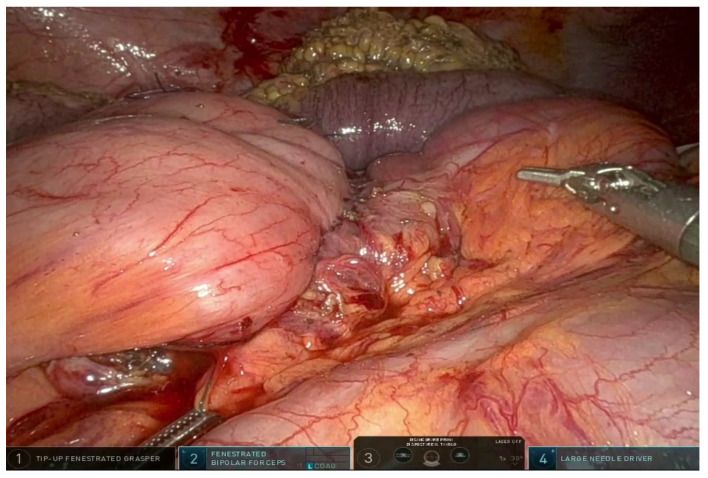
Intraoperative laparoscopic view showing small bowel loops during preparation for Kono-S anastomosis.

**Figure 2:**
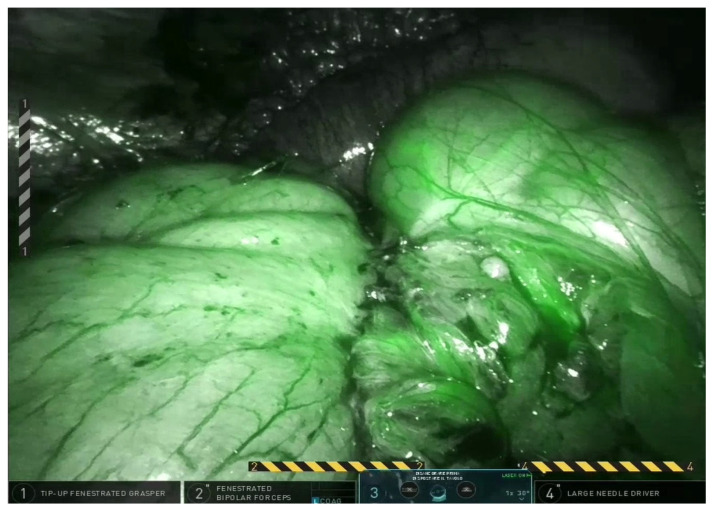
Indocyanine green (ICG) fluorescence imaging used intraoperatively to assess bowel perfusion before Kono-S anastomosis.

All patients underwent laparoscopic bowel resection followed by an extracorporeal Kono-S anastomosis, as previously described by Kono et al. [1]. The Kono-S technique involves the creation of a handsewn, antimesenteric, functional end-to-end anastomosis reinforced by a transverse supporting column. This configuration is designed to preserve luminal diameter, minimize mesenteric distortion, and reduce local inflammation at the anastomotic site. Laparoscopic surgery was performed in all cases (Figure 1 and Figure 2), as minimally invasive approaches have been associated with reduced postoperative pain, shorter hospital stay, and improved cosmesis in children [9]. The extracorporeal portion of the anastomosis was completed via a small abdominal incision, allowing precise hand-sewn construction while maintaining the benefits of laparoscopic visualization. Postoperative care followed standardized institutional protocols, including early mobilization, pain control, and monitoring for complications such as anastomotic leak or infection.

Anti-tumor necrosis factor (TNF)-alpha therapy was initiated postoperatively in all patients unless contraindicated [11]. Prophylaxis was typically started within 12 weeks, consistent with ECCO/ESPGHAN guidelines, with the goal of reducing the risk of early endoscopic and clinical recurrence [8]. Postoperative endoscopic surveillance was scheduled at 12 months for all patients, with earlier evaluation triggered by clinical symptoms such as abdominal pain, diarrhea, or weight loss [11]. This coordinated surgical–medical strategy aligns with contemporary recommendations emphasizing multidisciplinary care in pediatric Crohn’s disease.

### 2.4. Data Collection and Outcomes

Demographic and clinical data included age, sex, disease phenotype, surgical indication, anatomical site of resection, and timing of surgery (elective vs. urgent). Primary outcomes were postoperative complications and the feasibility of laparoscopic Kono-S anastomosis. Secondary outcomes included endoscopic recurrence assessed using the Rutgeerts score [12] at 12-month follow-up and timing of initiation of anti-TNF-alpha prophylaxis.

### 2.5. Statistical Analysis

Descriptive statistics were used to summarize demographic, clinical, and surgical variables. Continuous variables were reported as medians with ranges, and categorical variables were presented as counts and percentages. Given the small sample size and observational nature of the study, no inferential statistical tests were performed.

## 3. Results

### 3.1. Patient Characteristics and Surgical Indications

During the study period, a total of 11 pediatric patients with Crohn’s disease underwent bowel resection with a Kono-S anastomosis. The median age at the time of surgery was 14.7 years, ranging from 11 to 18 years, and the majority of patients were female (7/11, 63.6%). All patients presented with complicated disease phenotypes. Specifically, six patients exhibited stricturing disease, three had penetrating disease accompanied by abscess formation, and two demonstrated a combination of stricturing and penetrating features. The anatomical distribution of strictures varied: four patients had isolated ileal strictures, two patients had involvement of the ileocecal valve, and one patient presented with simultaneous strictures in both the ileum and cecum.

### 3.2. Surgical Approach

All surgical procedures were performed using a laparoscopic approach. Ten of the patients underwent an ileocolic resection, the standard procedure for ileocecal Crohn’s disease, while one patient required a small bowel-to-small bowel anastomosis due to a prior ileocecal resection. Among the eleven surgeries, nine were planned elective procedures, whereas two were conducted urgently in response to acute bowel obstruction. Importantly, no cases required conversion from laparoscopic to open surgery.

### 3.3. Postoperative Course and Complications

No postoperative complications were observed during the study period. The median hospital stay was 6.2 days. Throughout the study period, there were no reports of anastomotic leaks, surgical site infections, or the need for reoperation.

### 3.4. Follow-Up and Endoscopic Surveillance

The patients were followed for a median duration of 21 months, with a range of 3 to 35 months. Nine patients underwent follow-up ileocolonoscopy at the one-year mark, while two patients had not yet reached the 12-month timepoint at the time of data analysis. Among those evaluated, eight patients achieved a Rutgeerts score of 0, indicating complete endoscopic remission, and one patient had a score of 1, consistent with minimal endoscopic recurrence. All patients were initiated on postoperative anti-TNF-alpha prophylaxis after a median of 10.4 weeks (range 4–20 weeks).

Table 1 summarizes patient demographics, surgical indications, and outcomes.

## 4. Discussion

Ileocecal resection is the most common surgical procedure performed in pediatric Crohn’s disease, yet it is frequently followed by endoscopic and clinical recurrence [13], particularly at the anastomotic site. The development of anastomotic techniques that reduce the risk of recurrence is a critical objective, especially in young patients with a long disease duration ahead. Kono anastomosis, originally developed for adult patients (mainly those with Crohn’s disease), is increasingly being explored and applied in pediatric surgery, particularly in children with inflammatory bowel disease (IBD), such as Crohn’s. It is a specialised surgical technique for reconnecting the bowel after removing a diseased segment in children, with the same goal as in adults: to reduce postoperative recurrence, strictures, and improve long-term bowel function. Kono anastomosis may be considered in children, as Crohn’s children tend to have more aggressive or early onset of the disease, and traditional methods of anastomosis may have a higher frequency of recurrence and complications. The structural design of Kono anastomosis (the creation of a supporting column and the technique of hand-weaving) may better preserve the integrity and function of the bowel in growing children. Some considerations may be added as the technique is technically demanding and requires specialised surgical knowledge. Long-term data in paediatric patients are limited, and further studies are needed to confirm the long-term benefits, but the procedure seems particularly beneficial in patients with aggressive or early-onset Crohn’s disease.

To date, only two pediatric studies have assessed outcomes following Kono-S anastomosis [6,7]. In one study comparing Kono-S with traditional anastomoses, traditional anastomoses patients (n = 9) had longer lengths of stay (*p* = 0.03) and a significantly higher percentage of postoperative symptoms (*p* = 0.03) at 6 months in comparison to Kono-S patients (n = 9) [6]. Another retrospective study found the median endoscopic recurrence score at the anastomosis was 1 in 10 out of 12 pediatric CD patients 6 months after ileocecal resection with Kono-S anastomosis, with 6 of 10 patients being in endoscopic remission [7]. The results from our study align with and expand upon these findings. All patients underwent surgery laparoscopically, with no postoperative complications observed. At one-year follow-up, endoscopic recurrence was minimal, with only one patient having a Rutgeerts score above 0. Importantly, this is the first pediatric series to report endoscopic outcomes at the 12-month mark, providing critical insight into the medium-term effectiveness of the Kono-S technique in children. The role of early postoperative anti-TNF-alpha therapy should also be considered as a potential contributor to the favourable outcomes observed. The concurrent use of biologics represents a confounding factor that limits the ability to attribute the low recurrence rates exclusively to the Kono-S anastomotic technique. Controlled, prospective studies are needed to isolate the effect of the Kono-S technique from that of biologic prophylaxis and to determine the true additive or synergistic benefit of combining both strategies.

An important aspect when discussing pediatric outcomes is the unique disease trajectory in children with Crohn’s disease. Pediatric patients often present with more extensive and aggressive phenotypes, including upper gastrointestinal involvement and perianal disease, compared to adults [8]. The cumulative burden of repeated resections and the potential for impaired growth and pubertal delay underscore the need for surgical strategies that maximize bowel preservation. In this context, the Kono-S anastomosis is not merely a technical refinement but potentially a paradigm shift, as it aligns with the broader pediatric principle of minimizing intestinal loss and preserving function across a lifetime.

Our findings further reinforce that the Kono-S technique is feasible in a fully laparoscopic setting, which is particularly advantageous in pediatric populations. Minimally invasive surgery has consistently been associated with shorter recovery times, reduced postoperative pain, and improved cosmesis in children [9]. Importantly, performing a handsewn extracorporeal Kono-S does not appear to compromise the laparoscopic approach, suggesting that the procedure is adaptable even in smaller patients with limited intra-abdominal working space. This observation mirrors adult experiences where laparoscopic Kono-S has been demonstrated as safe and reproducible [14].

Another consideration is the role of mesenteric involvement in Crohn’s disease recurrence. Emerging evidence suggests that mesenteric inflammation and creeping fat are not passive bystanders but active drivers of disease recurrence [15]. By shifting the anastomosis to the antimesenteric border and incorporating a supporting column, the Kono-S technique may reduce the exposure of the anastomotic line to diseased mesentery. This theoretical advantage is particularly relevant in children, where the mesenteric immune environment may be especially dynamic and pro-inflammatory in the early years of disease. Future pediatric studies should consider incorporating mesenteric assessment, either radiologically or histologically, to evaluate whether the Kono-S truly modifies the natural history of recurrence via this pathway.

The absence of postoperative complications in our cohort is notable, given that pediatric Crohn’s resections are frequently performed in the setting of malnutrition, immunosuppression, or penetrating disease. Previous series have identified malnutrition, particularly hypoalbuminemia, as an independent predictor of postoperative complications in pediatric IBD [16]. While our small sample precludes firm conclusions, the favorable outcomes may reflect meticulous patient selection and perioperative optimization, but they also suggest that the technical aspects of Kono-S do not confer excess risk relative to conventional anastomoses. The absence of a contemporaneous control group limited statistical power, and the potential confounding effect of universal postoperative biologic prophylaxis restricts the ability to ascribe superiority to the Kono-S technique. These findings should therefore be interpreted cautiously and framed as hypothesis-generating. Larger datasets will be needed to confirm whether this safety profile holds across diverse clinical contexts.

One limitation of the current literature, including our own series, is the short to medium duration of follow-up. In adult cohorts, the durability of Kono-S has been demonstrated up to 5–10 years, with lower surgical recurrence rates compared to conventional techniques [2,3,4,5]. For pediatric patients, whose disease course spans decades, long-term validation is imperative. The present cohort contributes the longest median follow-up with endoscopic assessment at 12 months, which was missing in the prior pediatric reports. However, the median follow-up duration of 21 months is relatively short for a lifelong disease like CD. Ongoing longitudinal surveillance is necessary to assess the durability of remission and long-term recurrence rates, as well as growth, nutritional status, and quality of life. The establishment of multicenter pediatric Kono-S registries could accelerate the accrual of such long-term data. It should also be acknowledged that these outcomes may reflect institution-specific factors, including surgical expertise, postoperative care protocols, and access to multidisciplinary IBD services, which may limit generalizability to other centres.

Finally, our study contributes to the growing recognition that surgical innovation in pediatric IBD cannot be divorced from advances in medical therapy. The integration of optimized biologic prophylaxis with surgical strategies such as Kono-S may represent a “best of both worlds” approach, mitigating recurrence both mechanically and immunologically. This multimodal paradigm echoes recent ESPGHAN/ECCO consensus statements advocating for close coordination between surgeons and gastroenterologists in pediatric Crohn’s disease management [9]. Prospective trials stratifying outcomes by postoperative prophylaxis type (anti-TNF, anti-integrin, anti-IL-12/23) will be invaluable in clarifying the additive benefit of Kono-S.

While our study is limited by its retrospective design, single-centre nature, and small sample size (n = 11), it contributes to a growing but still limited literature base. Prospective multicentre studies with larger sample sizes and longer follow-up are necessary to determine whether the Kono-S technique should be preferentially adopted in pediatric CD surgical practice. Future directions should include the establishment of multicenter registries to aggregate long-term outcomes, standardized postoperative prophylaxis protocols to allow stratified analyses, and incorporation of patient-centered outcomes such as growth metrics, quality of life, and school attendance. Cost-effectiveness analyses may also support broader implementation.

## 5. Conclusions

Our experience suggests that Kono-S anastomosis is safe, feasible, and associated with encouraging early outcomes in pediatric Crohn’s disease. While superiority over conventional techniques cannot be established from this dataset, the absence of complications and the high rate of endoscopic remission at one year are promising. These findings should be viewed as preliminary evidence that justifies further prospective, multicenter studies. The absence of postoperative complications and low endoscopic recurrence at one year is encouraging. Kono-S may not only reduce the immediate risk of recurrence but also contribute to long-term bowel preservation, which is critical in children who may face multiple resections over their lifetime. By maintaining luminal integrity and reducing mesenteric stress at the anastomotic site, this technique may help preserve growth potential, nutritional status, and overall gastrointestinal function. Furthermore, the combination of Kono-S anastomosis with early postoperative anti-TNF-alpha therapy appears to offer a synergistic effect, reducing both clinical and endoscopic recurrence. While our study cannot fully separate the influence of biologics from the surgical technique, it provides preliminary evidence supporting an integrated surgical–medical approach.

From a broader perspective, these results have implications for surgical practice in pediatric IBD. Adoption of Kono-S may lead to shorter hospital stays, lower postoperative morbidity, and improved quality of life for children and their families. The technique’s compatibility with laparoscopic surgery also reinforces its feasibility in modern minimally invasive pediatric surgery programs.

While our findings are encouraging, they should be interpreted as hypothesis-generating due to the small sample size. Prospective multicenter studies with larger cohorts, longer follow-up, and standardized postoperative prophylaxis protocols will be essential to confirm the long-term benefits of Kono-S. Studies should also include patient-centered outcomes, such as growth parameters, nutritional status, psychosocial measures, and cost-effectiveness, to provide a comprehensive evaluation of its impact on pediatric Crohn’s disease management.

In summary, Kono-S anastomosis may represent a promising surgical innovation in pediatric Crohn’s disease. Our findings suggest its safety, feasibility, and potential efficacy in reducing postoperative recurrence, while highlighting the need for continued research to explore its role in long-term disease management. This approach may ultimately contribute to a paradigm shift in the surgical care of children with Crohn’s disease, emphasizing durable bowel preservation, reduced recurrence, and improved life-course outcomes.

## Figures and Tables

**Table 1:** Summary of key patient characteristics, surgical indications, and outcomes.

Variable	Total (n = 11)
Age, years, median (range)	14.7 (11–18)
Female, n (%)	7 (63.6)
Surgery type, n (%)	
Ileocolic resection	10
Small bowel-to-small bowel anastomosis *	1
Surgical indication, n (%)	
Stricturing disease	6
Penetrating disease	3
Both	2
Follow-up, months, median (range)	21 (3–35)
Rutgeerts score at one year, n (%)	
i0	10
i1	1
i2	0
i3	0
i4	0
Post-op anti-TNFα start, weeks, median (range)	10.4 (4–20)

* on a prior ileocecal resection.

## Data Availability

The original contributions presented in this study are included in the article. Further inquiries can be directed to the corresponding author.

## Abbreviations

The following abbreviations are used in this manuscript:

CD	Crohn’s disease
TNF	Tumor necrosis factor
